# Synthesis of Poly(2-aminothiazole)-Coated Polystyrene Particles and Their Excellent Hg(II) Adsorption Properties

**DOI:** 10.3390/polym12040749

**Published:** 2020-03-30

**Authors:** Hua Zou, Yiqian Wang

**Affiliations:** School of Materials Science and Engineering, University of Shanghai for Science and Technology, 516 Jungong Road, Shanghai 200093, China

**Keywords:** conjugated polymer, composite particles, poly(2-aminothiazole), adsorption, Hg(II)

## Abstract

Synthesis of conjugated polymer-coated latex particles is an effective method to improve the poor processability of conjugated polyheterocycles. The key to success is to control the overlayer thickness so it is less than the size of the solvated layer of polymeric stabilizer. This paper presents a protocol to coat polymer latex particles with poly(2-aminothiazole) (PAT), which is a relatively new heterocyclic conjugated polymer. The protocol is based on chemical oxidative polymerizations of 2-aminothiazole using copper chloride as the oxidant at a fixed oxidant/monomer molar ratio of 0.5 in aqueous media in the presence of poly(*N*-vinyl-2-pyrrolidone)-functionalized polystyrene (PS) latex. The effects of monomer concentration, PS concentration, and polymerization temperature on the morphology of the PAT-coated PS composite particles were investigated by SEM and TEM, and the resulting composite particles characterized by FTIR and XPS. Optimization of the initial monomer concentration allowed colloidally stable PAT-coated PS composite particles to be formed at ambient temperature, and the PAT loading was easily adjusted by varying the initial PS concentration. The Hg(II) adsorption properties of selected PAT-coated PS composite particles were assessed preliminarily. The maximum adsorption capacity at 25 °C reached 440.25 mg/g, which is much higher than many other adsorbents.

## 1. Introduction

Conjugated polymers typically involve polyheterocycles, such as polypyrrole (PPy), polyaniline (PANI), and poly(3,4-ethylenedioxythiophene) (PEDOT), which can be synthesized by electrochemical or chemical oxidative polymerization methods. Compared with the electrochemical method, the chemical method has several advantages in terms of massive production, facile reaction conditions and simple doping chemistry. However, such a method suffers from an obvious disadvantage: the processability of the product, in the form of bulk powder, is normally poor due to the strong interchain interactions and crosslinking. To address this problem, several routes for the synthesis of conjugated polymers in the form of colloids have been developed [1]. These typically include: (i) sterically-stabilized polypyrrole latexes [2]; (ii) conjugated polymer-silica colloidal nanocomposites [3,4,5]; and (iii) conjugated polymer-coated latex particles [6,7,8]. Among them, the principle of the formation of conjugated polymer-coated latex particles is as follows: when the conjugated polymer is formed as an ultrathin layer at the surface of the sterically stabilized latex particles, the colloidal stability of the conjugated polymer-coated latex particles can be maintained if the overlayer thickness is less than the size of the solvated layer of polymeric stabilizer. A series of conjugated polymer-coated latex particles based on PPy, PANI, and PEDOT has been prepared by chemical oxidative polymerization in the presence of preformed latex particles, respectively [1].

Poly(2-aminothiazole) (PAT) is a relatively new heterocyclic conjugated polymer that can be polymerized from 2-aminothiazole (AT) by the chemical oxidative method with various oxidant/solvent systems [9,10,11,12,13,14,15,16,17]. Recently, we reported the preparation of PAT by the chemical oxidative method using copper chloride as an oxidant in aqueous solution without adding any acid [13]. The adsorption capability of PAT for Hg(II) in the aqueous solution was also evaluated. In this context, we note that Hg(II) is one of the most toxic heavy metal ions in existence, and adsorption is considered to be one of the most attractive processes used for Hg(II) removal since adsorption is easy and cost-effective when compared to other techniques, such as ion exchange, solvent extraction, and precipitation [18,19,20,21,22]. On the other hand, heterocyclic conjugated polymers are used as heavy metal ion adsorbents because these polymers typically carry sulfur- or nitrogen-containing functional groups, which have a high affinity for heavy metal ions such as Hg(II) [23]. Notably, AT has a much higher sulfur and nitrogen atom molar ratio (30%) than that in 3,4-ethylenedioxythiophene (7%), aniline (7%), and pyrrole (10%). However, analogous to polyaniline and polypyrrole, PAT prepared by the chemical oxidative method is typically aggregated, which means there is a relatively low surface area available for adsorption. One elegant solution to this problem is to synthesize conjugated polymer-coated latex particles, which should have a reasonably higher amount of conjugated polymer on the surface. Therefore, compared with powdered PAT, PAT-coated latex particles should have a higher specific surface area and thus can benefit the adsorption process. 

**Figure 1:**
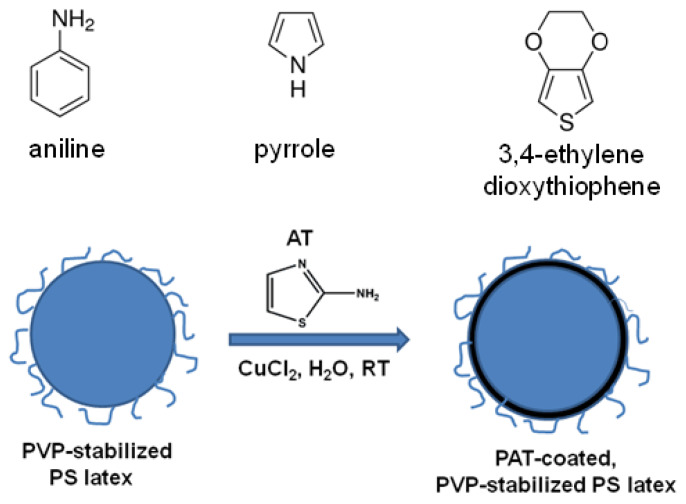
Chemical structures of aniline, pyrrole, 3,4-ethylenedioxythiophene, and 2-aminothiazole, and a scheme for the formation of poly(2-aminothiazole) (PAT)-coated poly(*N*-vinyl-2-pyrrolidone)-functionalized polystyrene (PS) latex particles.

In the present work, we report on the deposition of PAT onto near-monodisperse, micrometer-sized PS latexes based on chemical oxidative polymerizations of AT in aqueous media in the presence of poly(*N*-vinyl-2-pyrrolidone) (PVP)-functionalized polystyrene (PS) latex (see Figure 1). PS is a frequently used “model” polymer in such studies since it is readily synthesized with high monodispersity at different sizes, and its morphology is particularly suitable for electron microscopy observation due to its relatively high *T*_g_. It is perhaps noteworthy that AT has a relatively high water solubility (100 g/L), much higher than that of pyrrole (60 g/L), aniline (36 g/L), and 3,4-ethylenedioxythiophene (immiscible with water), suggesting AT may be well-suited for such preparation. The effects of monomer concentration, PS concentration, and polymerization temperature on the morphology of the composite particles were investigated. Furthermore, the adsorption properties of the PAT-coated PS (PAT-PS) latexes for Hg(II) in aqueous solution were assessed preliminarily. As far as we are aware, this is the first work on the synthesis of PAT in the form of conjugated polymer-coated latexes, and the adsorption properties of the PAT-coated PS composite particles, which exhibit relatively high adsorption capacity, are reported for the first time.

## 2. Materials and Methods 

### 2.1. Materials

Styrene, AT (97%), PVP (*M*_w_ = 360,000), and azoisobutyronitrile were purchased from Sigma-Aldrich (St. Louis, MO, USA). 2-Propanol (>99.5%) was purchased from TCI (Shanghai, China) Development Co., Ltd. CuCl_2_∙2H_2_O (99%) was obtained from Sinopharm Chemical Reagent (Shanghai, China). The styrene monomer was purified by passing it through a basic aluminum oxide column. All other chemicals were used as received.

### 2.2. Preparation of PVP-Functionalized PS Latexes 

The monodisperse PS latex particles were prepared by dispersion polymerization as follows: 10 g of styrene, 1.4 g of PVP, and 80 g of 2-propanol were charged into a 250-mL three-neck flask equipped with a mechanical stirrer, an N_2_ inlet, a Graham condenser, and a heating mantle. The solution was stirred and purged N_2_ for 30 min and then heated to 70 °C. A solution of AIBA (100 mg) dissolved in 10 g of H_2_O was added to initiate the polymerization, which was allowed to proceed for 24 h at 70 °C under N_2_. The product was then purified by repeated centrifugation-redispersion cycles, with each supernatant being replaced with deionized water. Finally, the particles were redispersed in deionized water, giving a solid content of 4.6 wt%.

### 2.3. Preparation of PAT-PS Particles 

A typical synthesis of PAT-coated PS particles was conducted as follows: 8.7 g of the PS latex (equivalent to 0.4 g of dry PS particles), 0.2 g (2 mmol) of 2-aminothiazole, and 26.7 of H_2_O were charged into a 100 mL flask containing a magnetic stir bar. A solution of CuCl_2_∙2H_2_O (0.172 g, 1 mmol) in deionized water (5.0 g) was then added dropwise to the stirring solution. In each synthesis, the oxidant/monomer ratio was fixed to be 0.5, and the total water content was fixed to be 50 g. The color of the dispersion changed immediately after the CuCl_2_ solution was added. The reaction was allowed to proceed for 24 h at room temperature (~25 °C) unless otherwise stated. The resulting dispersion was purified by repeated centrifugation-redispersion cycles, with each supernatant being replaced with deionized water.

### 2.4. Characterization 

The sizes and size distributions of the latex particles were determined by Dynamic light scattering (DLS) using a Malvern Mastersizer 2000 instrument. Both scanning electron microscopy (SEM, Quanta FEG 450, FEI, Hillsboro, OR, USA), and transmission electron microscopy (TEM, Tecnai G2 F30, FEI, Amsterdam, Netherlands) were used to observe the morphology of the particles. The SEM sample was prepared by placing several drops of purified latex onto an adhesive carbon disk mounted on an aluminum stub. After drying under ambient conditions overnight, the stub was then sputter-coated with a thin layer of gold prior to examination at 30 kV. The TEM sample was prepared by drying a drop of purified latex on a carbon-coated copper grid prior to examination at 300 kV. A PerkinElmer Spectrum100 FT-IR spectrometer was used to collect the FTIR spectra. Surface compositions of the particles were characterized with an X-ray photoelectron spectroscope (XPS, Versa Probe PHI-5000, ULVAC-PHI Inc., Kanagawa, Japan).

### 2.5. Adsorption Experiments

Ten milligrams of dried PAT-PS particles (prepared in run 5) were charged into a conical flask with 25 mL of Hg(II) solution at different initial concentrations (100–600 mg/L), with the pH having been adjusted to 4.5 prior to mixing. The samples were stirred at 280 rpm at 25 °C for 24 h followed by filtration. The equilibrium concentration of Hg(II) in the filtrate was then determined by inductively coupled plasma atomic emission spectrometry (ICP-AES). The adsorption capacity (*Q*, mg/g) was calculated according to the following equation [13]:

(1) $$Q=\frac{\left(C_{0}-C_{e}\right)}{W}V$$

where *C*_0_ is the initial concentration of Hg(II) solution (mg/L), *C*_e_ is the equilibrium concentration of Hg(II) solution (mg/L), *V* is the solution volume (mL), and *W* is the mass of PAT-PS particles (mg). The adsorption kinetics experiment for Hg(II) was carried out with 20.0 mg of dried PAT-PS particles and 50 mL of Hg(II) solution at an initial concentration of 500 mg/L.

## 3. Results and Discussion

**Figure 2:**
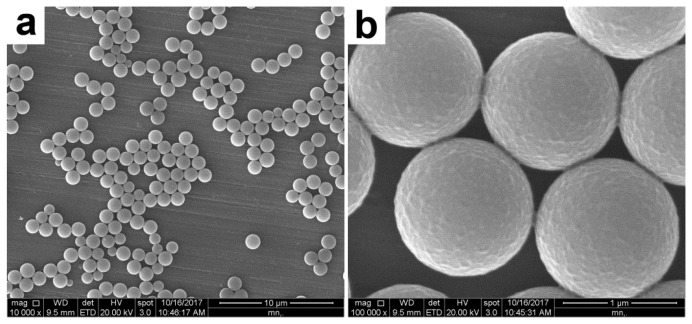
TEM images of PVP-stabilized PS particles at (**a**) lower and (**b**) higher magnifications.

**Figure 3:**
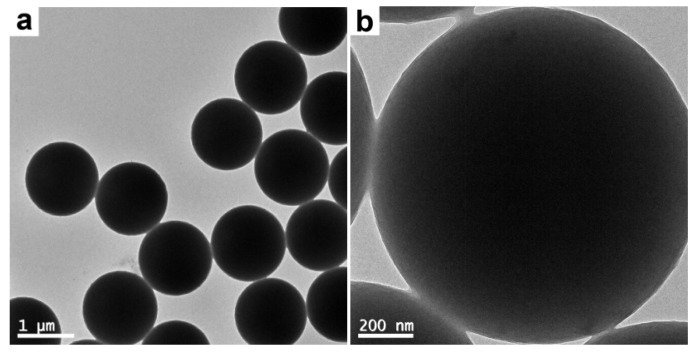
TEM images of PVP-stabilized PS particles at (**a**) lower and (**b**) higher magnifications.

Near monodisperse PS template particles (see Figure 2 and Figure 3) with an average diameter of ~1.17 μm as determined by DLS were first prepared by dispersion polymerization using PVP as the stabilizer. This frequently-used steric stabilizer can be grafted onto the latex particles during the dispersion polymerization process [24]. As confirmed by XPS (the S signal as a marker for PVP, not shown), the PS particles were functionalized by PVP and incorporated hydrophilic PVP on their surfaces. 

The PS particles were then subjected to the coating process under the conditions that PAT could be formed. More specifically, the syntheses were conducted using copper chloride as the oxidant and 2-aminothiazole as the monomer in aqueous media in the presence of purified PS latex at a fixed oxidant/monomer molar ratio of 0.5. This ratio was selected to ensure approximately spherical PAT particles were formed, since our previous experiments indicated that PATs prepared at a ratio higher or lower than 0.5 were less spherical [5], which was unfavorable for the formation of homogenous coating.

A series of PAT-coated PS particles was prepared under different conditions, as summarized in Table 1. The colloidal stability of the PAT-coated PS particles is the primary concern of this study. According to the principle of the formation of conjugated polymer-coated latex particles, colloidally stable PAT-coated particles will be formed only if the thickness of the PAT overlayer is within the size range of PVP, which is approximately 20–30 nm [25]. In this study, the colloidal stability of the composite particles was monitored by visual inspection; the surface roughness of the composite particles was examined by SEM and TEM observations; and the thickness of the PAT overlayer was determined from high-magnification TEM images utilizing the subtle contrast between the PS particles and the PAT coatings.

### 3.1. Effect of the AT Concentration 

**Figure 4:**
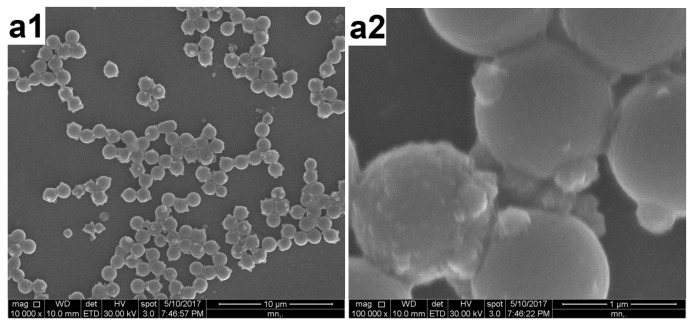

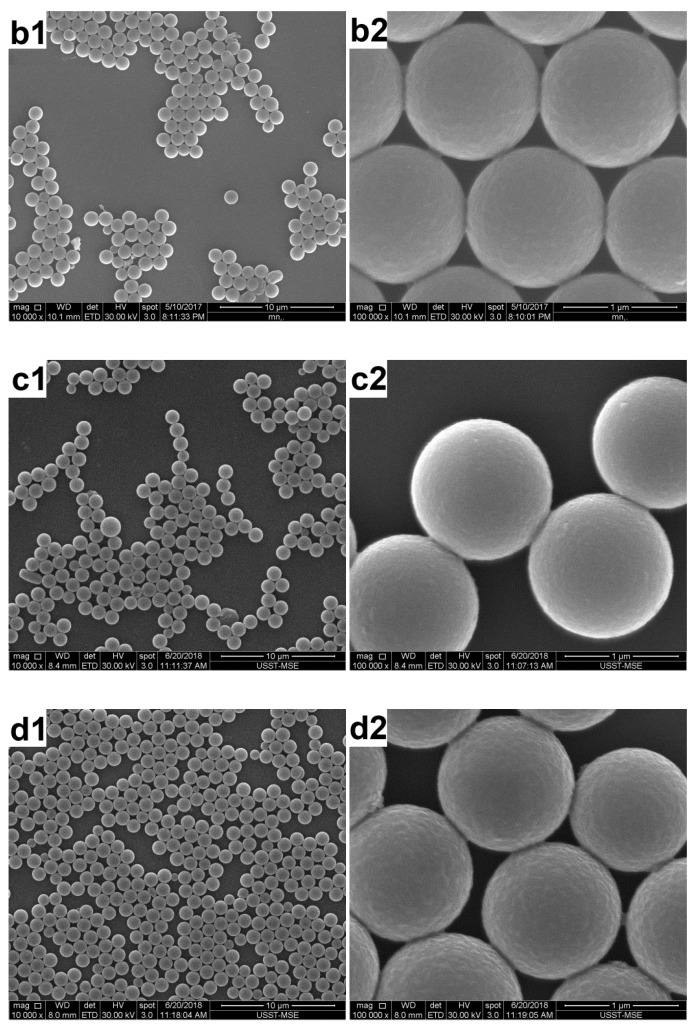
SEM images of the products prepared with a fixed amount of PS (0.4 g), and varied AT amounts at various magnifications: (**a**) 0.4 g (run 1); (**b**) 0.3 g (run 2), (**c**) 0.2 g (run 3); and (**d**) 0.1 g (run 4).

**Figure 5:**
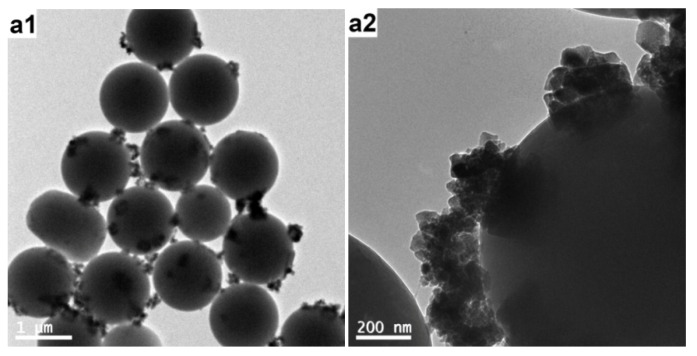

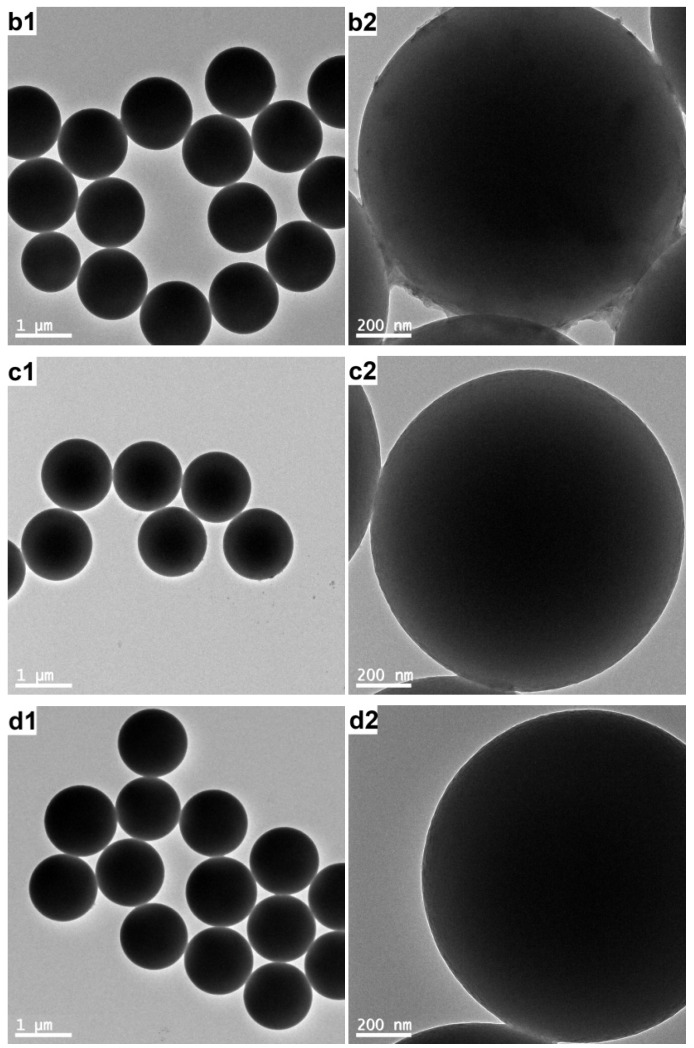
TEM images of the products prepared with a fixed amount of PS (0.4 g), and varied AT amounts at various magnifications: (**a**) 0.4 g (run 1); (**b**) 0.3 g (run 2), (**c**) 0.2 g (run 3); and (**d**) 0.1 g (run 4).

In the first set of experiments, the effect of the AT concentration on the morphology of the composite particles was studied. Figure 4 and Figure 5 show the SEM and TEM images of the products prepared at a fixed amount of PS (0.4 g), and varied AT amounts, respectively. Obviously, the colloidal stability of the final products depended on the amount of AT present. The polymerization of 0.4 g of AT led to macroscopic precipitation of the system. This was probably because the polymerization rate was too high at this monomer concentration, as the rate of the polymerization reaction increased with increasing monomer concentration [14]. As a result, in addition to PS particles with a thick PAT coating, separate PAT subphases with a morphology similar to that of the PAT bulk powder were clearly observed (see Figure 4a and Figure 5a). These PAT subphases should also be responsible for the loss of colloid stability by acting as a bridging flocculant between the latex particles. When 0.3 g of AT was used for the polymerization, substantial flocculation was observed while redispersion was possible by mechanical shaking. The product showed relatively rough surfaces (see Figure 4b and Figure 5b), in contrast to the smooth surfaces of the original PS particles. With 0.2 and 0.1 g of initial AT, the products were colloidally stable. However, the presence of the PAT overlayer was not visible even with high-magnification SEM (Figure 4c,d) and TEM (see Figure 5c,d) studies, which showed the coated particles were essentially identical to the original PS particles. This was consistent with the earlier studies on PPy-coated PS particles, in which the PPy overlayer was very thin, smooth, and uniform [6].

### 3.2. Effect of the PS Concentration 

**Figure 6:**
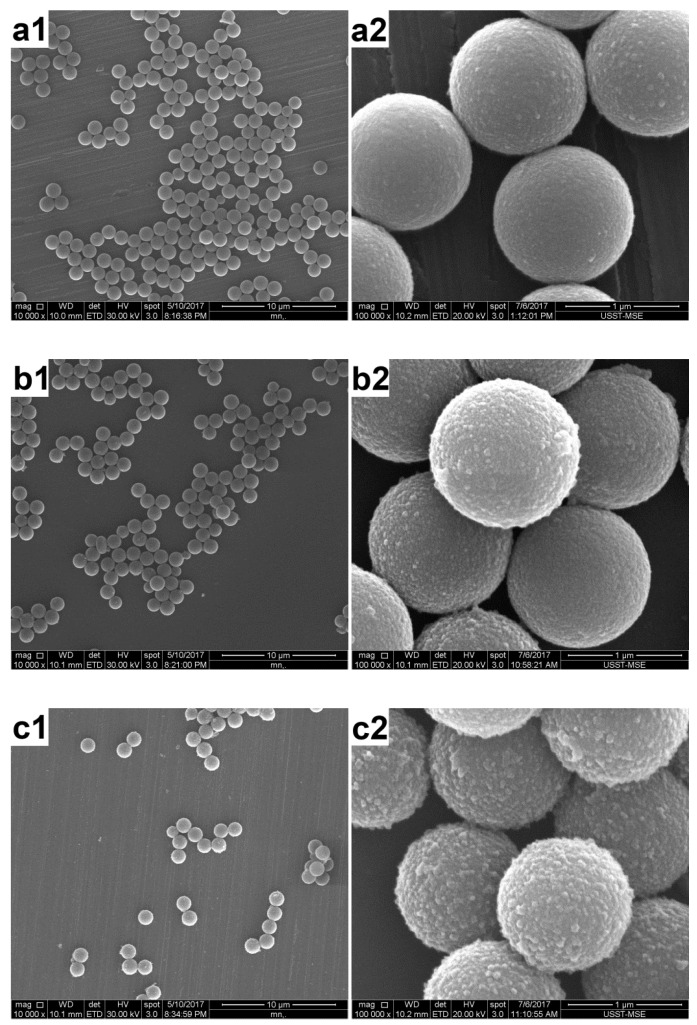
SEM images of the products prepared with varied PS amounts and a fixed amount of AT (0.2 g) at various magnifications: (**a**) 0.2 g (run 5); (**b**) 0.1 g (run 6), and (**c**) 0.05 g (run 7).

**Figure 7:**
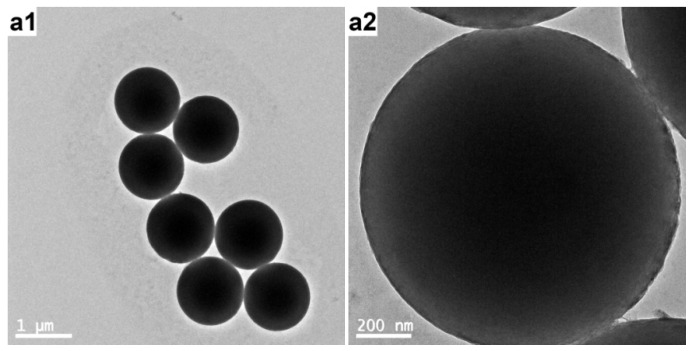

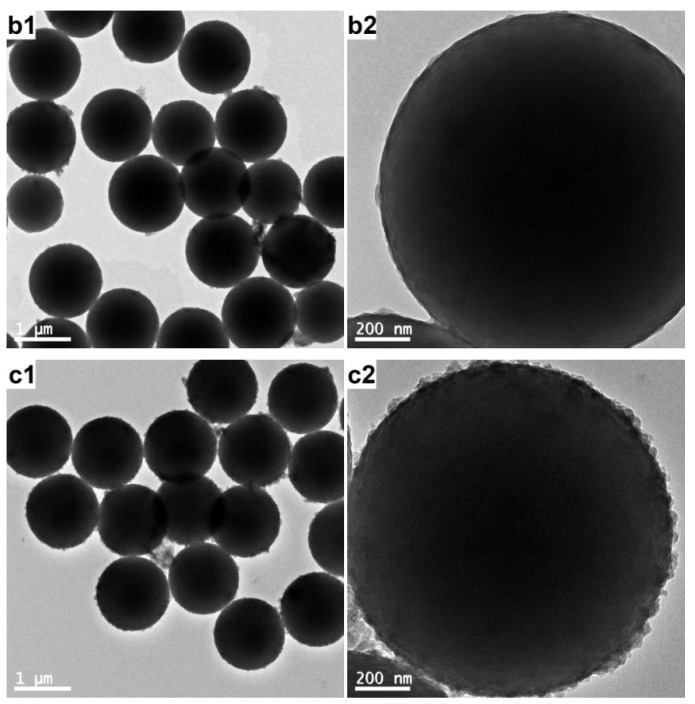
TEM images of the products prepared with varied PS amounts and a fixed amount of AT (0.2 g) at various magnifications: (**a**) 0.2 g (run 5); (**b**) 0.1 g (run 6), and (**c**) 0.05 g (run 7).

**Figure 8:**
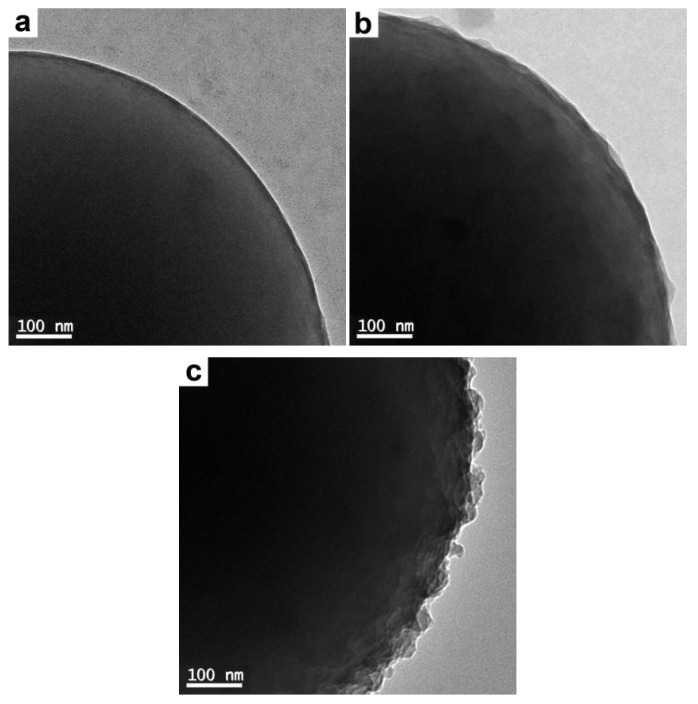
TEM images of the products prepared with varied PS amounts and a fixed amount of AT (0.2 g) at higher magnifications: (**a**) 0.2 g (run 5); (**b**) 0.1 g (run 6), and (**c**) 0.05 g (run 7). The overlayer thickness of the coated particles was measured to be ~7, ~17, and ~28 nm, respectively.

To prepare a series of composite latexes with various PAT loadings, the polymerizations were also conducted with a fixed monomer concentration (the oxidant/monomer ratio was fixed to be 0.5) and varied PS concentrations. Considering that a higher monomer concentration possibly caused bigger PAT granules as mentioned previously, and a lower monomer concentration led to extremely low yields of PAT, the amount of monomer was fixed to be 0.2 g. The corresponding SEM and TEM images are shown in Figure 6, and Figure 7 (in combination with Figure 4c, and Figure 5c), respectively. It is obvious that the PAT coating became rougher with decreasing PS concentration. For example, particles with extremely thin PAT coatings were obtained when using higher amounts of PS latex particles (0.4 and 0.2 g, see Figure 3c and Figure 6a, respectively). When 0.1 and 0.05 g of PS latex particles were used, the existence of PAT coatings was clearly visible from SEM images (see Figure 6b,c, respectively). This could be attributed to the decreasing numbers of PS particles available for PAT deposition, which meant each PS particle would be coated by more PAT granules in principle [6]. The overlayer thickness of the coated particles prepared with 0.2, 0.1, and 0.05 g of PS was ~7, ~17, and ~28 nm, respectively, as measured from their magnified TEM images (Figure 8). It should be noted the overlayer thickness of the coated particles prepared with 0.4 g of PS was too thin to be accurately measured (see Figure 5c), but should be less than 7 nm. Moreover, the system prepared with 0.05 g of PS latex particles was not very stable on standing. This could be best explained by the fact that the overlayer thickness was ~28 nm, which was closer to the upper limit of the size of the stabilizer [25]. Thus the thickness of these PAT coatings could be simply controlled by tuning the amount of PS latex particles used for PAT deposition.

### 3.3. Effect of the Polymerization Temperature

**Figure 9:**
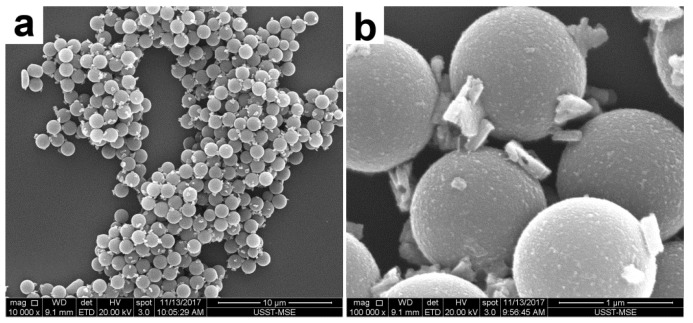
SEM images of the products prepared at 50 °C (run 8) at (**a**) lower (10,000×) and (**b**) higher (100,000×) magnifications.

PAT deposition was also attempted at higher temperatures (50 and 70 °C, runs 8 and 9, respectively) under other identical conditions of the typical synthesis (run 3). However, this protocol was unsuccessful and macroscopic precipitation was observed during the polymerization process. Figure 9 displays the SEM images of the sample prepared at 50 °C. This was similar to the case of using a higher amount of AT (run 1) [14]; that is, the polymerization rate was very high, leading to the formation of a large amount of separate PAT subphases. Thus, a higher polymerization temperature was unfavorable for the formation of a uniform overlayer. 

### 3.4. Characterization of the PAT-PS Particles

**Figure 10:**
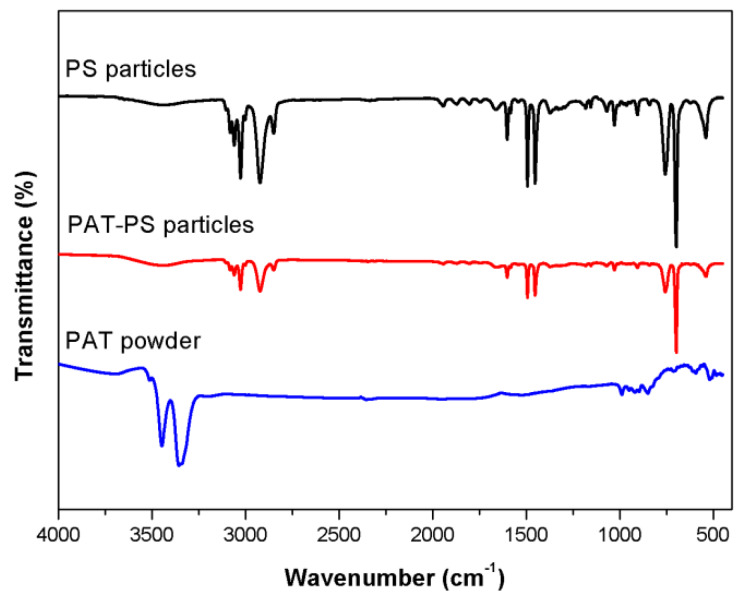
FTIR spectra of PAT (prepared at an oxidant/monomer ratio of 0.5), PS, and typical PAT-PS particles (run 4). Note the relatively weak bands due to the PAT component in the spectrum of PAT-PS particles relative to the spectrum of PS particles.

The FTIR spectra of PAT, PS, and typical PAT-coated PS particles (run 4) are shown in Figure 10. For the spectrum of the uncoated PS particles, the main peaks at 1493, 1452, 757, and 698 cm^−1^ are typical for that of PS, and an additional peak at 1663 cm^−1^ is attributable to the pyrrolidone carbonyl of the PVP stabilizer [6]. The FTIR spectrum of the PAT-coated PS composite particles exhibits similar bands assignable to PS components, while the intensity of the peaks appears to be relatively weak compared to those of original PS particles, indicating the possible effect of PAT coating on the PS particles. Meanwhile, the band assignable to PAT (3356 cm^−1^, stretching of the secondary –NH groups [17]) is not obvious, given the extremely low PAT loading. 

**Figure 11:**
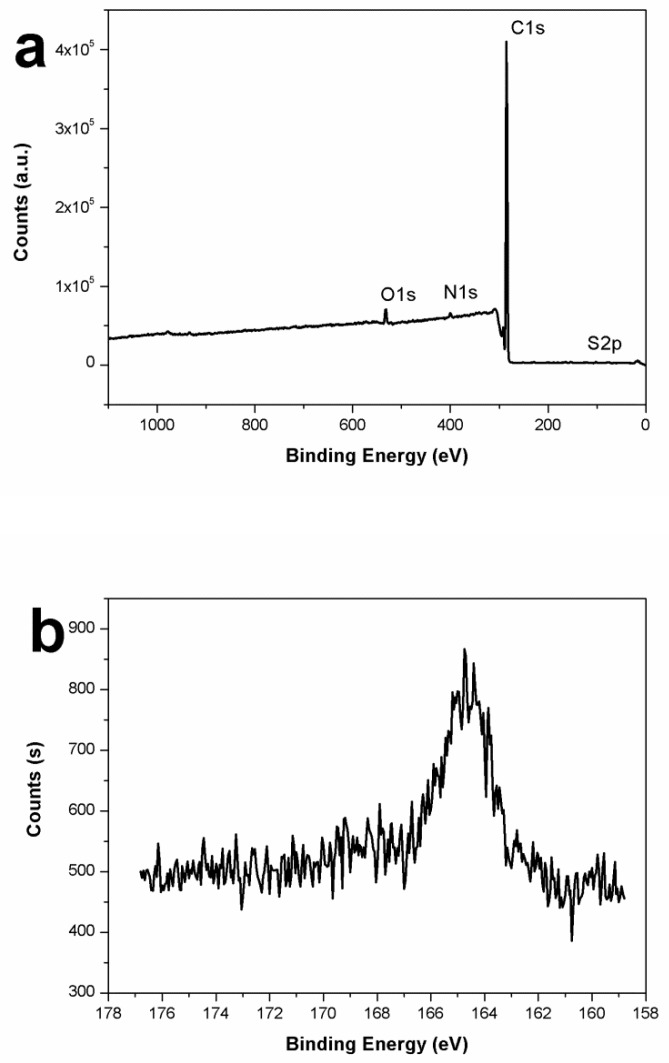
XPS spectra of typical PAT-PS particles (run 4): (**a**) total spectrum; and (**b**) S 2p spectrum.

To further confirm the successful coating of PAT onto PS particles, the PAT-PS particles were characterized with XPS, which is a technique well-suited to analyzing the surface compositions of colloidal particles with a typical sampling depth of 2–10 nm [24]. The sample prepared under the conditions of PS amount of 0.4 g and AT amount of 0.2 g, whereby the presence of the PAT overlayer was not visible by SEM, was selected to demonstrate the effectiveness of XPS. The S signal can be used as a unique marker for PAT. As shown in Figure 11, the presence of PAT on the particle surface was evidenced from the peak at 164.8 eV (S 2p), albeit at relatively low concentration. Furthermore, the PAT coverage percentage on the PAT−PS particle surface could be estimated from the XPS result using the following equation [24]:

% surface PAT = (% surface S) × 100/(% S of the PAT)(2)

where the % surface, S, obtained from the XPS data was 0.28 % and the % S of the PAT (C_3_H_4_N_2_S) was 32%. Thus, the % surface PAT of the PAT−PS particles was calculated to be 0.875%. Similarly, the PVP coverage percentage on the PAT−PS particle surface could be estimated as follows:

% surface PVP = (% surface N contributed by PVP) × 100/(% N of the PVP)(3)

where the % surface, N, contributed by PVP could be obtained from the XPS data (1.125%), and the % N of the PVP (C_6_NO) was 15.7%. Thus, the % surface PVP of the PAT−PS particles was determined to be 7.2%. These values were in reasonable agreement with the ultrathin overlayer in this case.

### 3.5. Adsorption Properties of the PAT-PS Particles

**Figure 12:**
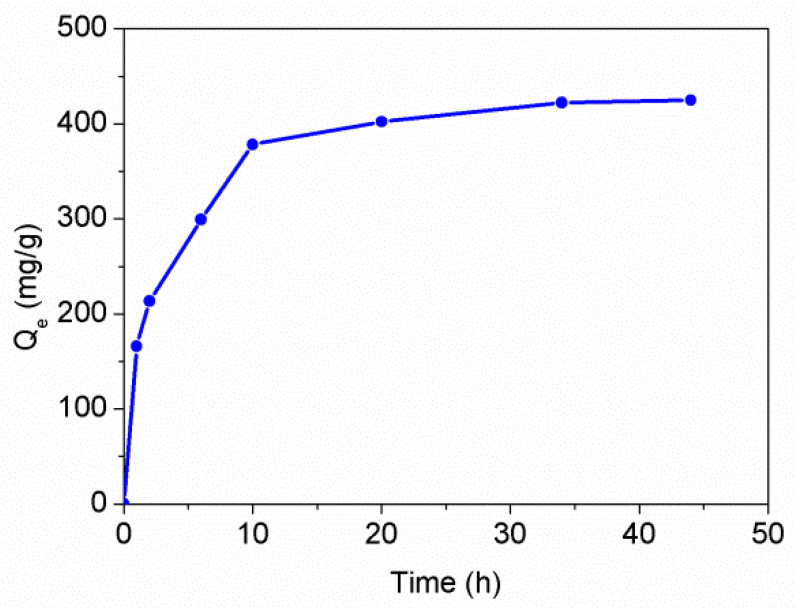
The adsorption kinetics behavior of PAT-PS particles for Hg(II) in aqueous solution (PAT-PS 20 mg, *C*_0_ = 500 mg/L, pH 4.5).

The feasibility of the PAT-PS particles as an Hg(II) remover in aqueous solution was explored. The PAT-PS particles prepared with 0.2 g of PS and 0.2 g of PAT (run 5) were selected for adsorption study as they had a reasonable thickness of PAT coating (~7 nm) and higher product mass for adsorption. To optimize the adsorption process, the effect of the contact time on the adsorption capacity of PAT-PS particles for Hg(II) was studied first. As shown in Figure 12, the adsorption capacities increased substantially within the initial 2 h, and then increased steadily with increasing contact time up to 24 h. Subsequently, the adsorbed amount remained almost constant. This behavior was very similar to that of the neat PAT [13]. Thus, we can assume the adsorption reached equilibrium within 24 h.

**Figure 13:**
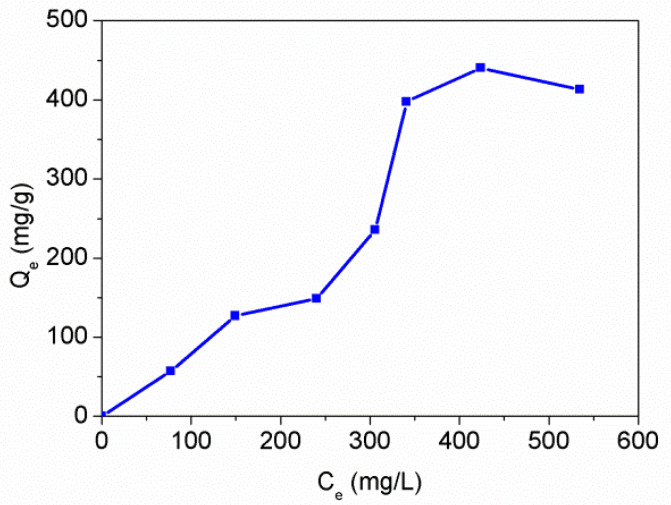
Adsorption isotherm of PAT-PS particles for Hg(II) in aqueous solution (PAT-PS 10 mg, *C*_0_ = 100–600 mg/L, pH 4.5, contact time 24 h).

Subsequently, the adsorption isotherm was obtained with a contact time of 24 h (see Figure 13). Significant deviations from Langmuir-type of adsorption isotherm were observed, suggesting a complex adsorption mechanism. In previous work, we suggested that the adsorption of Hg(II) to PAT was likely attributed to complexation between the ions and the exocyclic N and S atoms of PAT, while the endocyclic nitrogen was not involved in the adsorption [13]. The Hg(II) adsorption capacity of various adsorbents has been summarized in two recent reviews [26,27], and the maximum adsorption capacities for Hg(II) ions with several various adsorbents are compared in Table 2. It is shown that the adsorption capacity (440.25 mg/g at 25 °C) obtained with PAT-PS particles in this work was much higher than that of many other adsorbents reported in literature. For example, the maximum adsorption capacity of a PAT/CA fiber membrane was only 177 mg/g at 25 °C [15]. The excellent adsorption properties of the PAT-PS particles for Hg(II) should be primarily ascribed to their having a high surface area available for adsorption.

## 4. Conclusions

We describe a facile and effective protocol for coating micrometer-sized, sterically stabilized PS latex particles with PAT overlayers. Colloidally stable PAT-PS particles can be obtained at a proper AT concentration, and the PAT loading can be easily adjusted by varying the initial PS particle concentration. At low PS concentrations, the overlayer appeared to be reasonably smooth and uniform; but at high concentrations, increasing overlayer roughness was apparent. The PAT-PS particles exhibited excellent adsorption properties for Hg(II) in aqueous solution due to their having a high surface area available for adsorption.

## Figures and Tables

**Table 1:** Summary of the PAT-coated PS particles prepared under different conditions. The total water content was fixed to be 50 g.

Run	Initial PS Mass (g)	Initial AT Mass (g)	Temperature (°C)	Remarks
1	0.4	0.4	25	precipitation
2	0.4	0.3	25	flocculation
3	0.4	0.2	25	stable, smooth
4	0.4	0.1	25	stable, smooth
5	0.2	0.2	25	stable, smooth
6	0.1	0.2	25	stable, rough
7	0.05	0.2	25	metastable, rough
8	0.4	0.2	50	precipitation
9	0.4	0.2	70	precipitation

**Table 2:** Comparison of maximum adsorption capacity of Hg(II) ions with various adsorbents.

Adsorbent	Maximum Adsorption Capacity (mg/g)	Ref.
PAT	325.7 mg/g at 308 K	[13]
PAT/cellulose acetate fiber membrane	177 mg/g at 298 K with 6.5 wt % PAT	[15]
AT-functionalized polyacrylonitrile	454.9 mg/g at 308 K	[28]
thiol-functionalized mesoporous silica	47.50 mg/g at 293 K	[29]
starch/SnO_2_ nanocomposite	192 mg/g at 298 K	[30]
PAT-coated PS particles	440.25 mg/g at 298 K	this work
